# Microcytic Anemia: An Insidious Presentation of Sickle Cell Beta+ Thalassemia, a Rare Sickle Cell Variant

**DOI:** 10.7759/cureus.23293

**Published:** 2022-03-18

**Authors:** Malavika Shankar, Nicole Gousy, Tutul Chowdhury

**Affiliations:** 1 Internal Medicine, Interfaith Medical Center, New York City, USA; 2 Medicine, American University of Antigua, New York City, USA; 3 Internal Medicine, One Brooklyn Health System, Brooklyn, USA

**Keywords:** sickle cell disease: scd, microcytic hypochromic anemia, beta thalassemia, adult sickle cell anemia, sickle cell beta-thalassemia

## Abstract

Sickle cell disease variants can commonly present as life-threatening complications, like sequestration crisis, hypersplenism, or stroke. However, clinicians should also look for milder findings like asymptomatic chronic anemia mimicking iron deficiency as a milder, more insidious clue to an underlying sickle cell variant. Early investigations of these milder symptoms can potentially reduce the risk of more severe complications such as vaso occlusive crisis. In this report, we present a 75-year-old African-American female, who was referred to the hematology clinic for chronic anemia without any history of vaso occlusive crisis and was eventually diagnosed with sickle cell beta plus thalassemia as per hemoglobin electrophoresis. Here, we review the challenges in diagnosing rarer types of sickle cell disease and the importance of educating patients about the diagnosis. This rare type demands clinicians' awareness to identify the disease early and to understand the etiology of the complications, if any, that occur.

## Introduction

Sickle cell disease (SCD) is a genetically predisposed hemoglobinopathy with an approximate prevalence of 100,000 in the United States which could involve multiple systems, particularly when not optimally treated. SCD exhibits high mortality and morbidity and typically patients will show signs of extensive cerebrovascular involvement due to the pathogenesis of this disease [1,2]. S/β+ type of SCD, also known as sickle cell trait beta thalassemia trait, is one of the rarer genotypes of SCD that may present with no symptoms or possibly mild anemia, but eventually will develop life-threatening complications such as avascular necrosis, retinopathy, and potentially strokes [2-4]. Due to its silent and gradual progression, this rare type might stay undiagnosed contributing to its sudden detrimental clinical outcome in adulthood [5,6]. Herein we report a 75-year-old African-American female with SCD (S/β+ type) without any related symptoms endorsed, and treated as iron-deficiency anemia at the very beginning without any considerable improvement. The lack of substantial improvement instigated further investigation leading to the eventual diagnosis of Sickle cell beta plus thalassemia established through hemoglobin electrophoresis.

## Case presentation

We report a 75-year-old African-American female with a past medical history of chronic kidney disease, hypertension, hyperlipidemia, who was referred from her primary care physician for evaluation of chronic microcytic anemia. 

**Figure 1:**
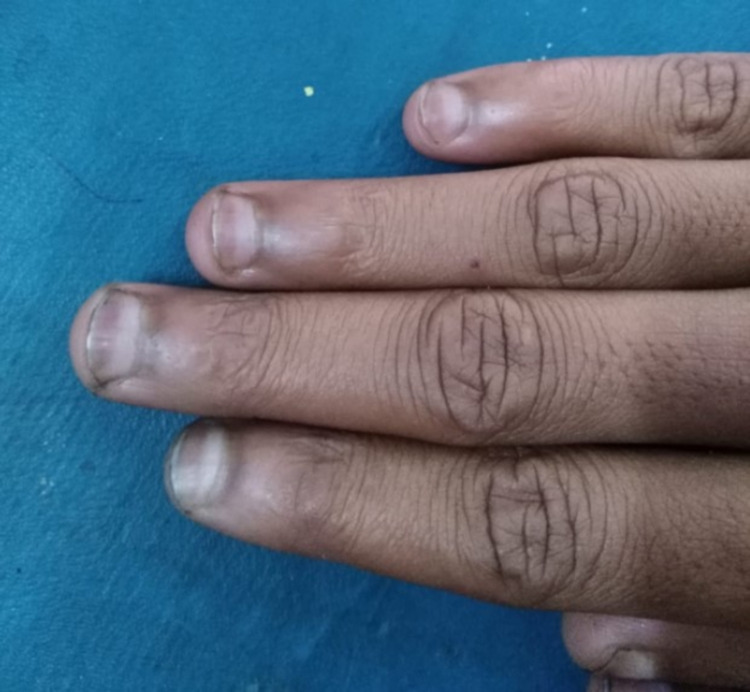
This image highlights the “half and half” or “Lindsay's nails,” which are characterized by a white discoloration of the proximal portion of the nail along with the distal half red, pink, or brown, with a sharp line of demarcation between the two portions. This is a nonspecific finding however and was found on all 10 digits.

**Figure 2:**
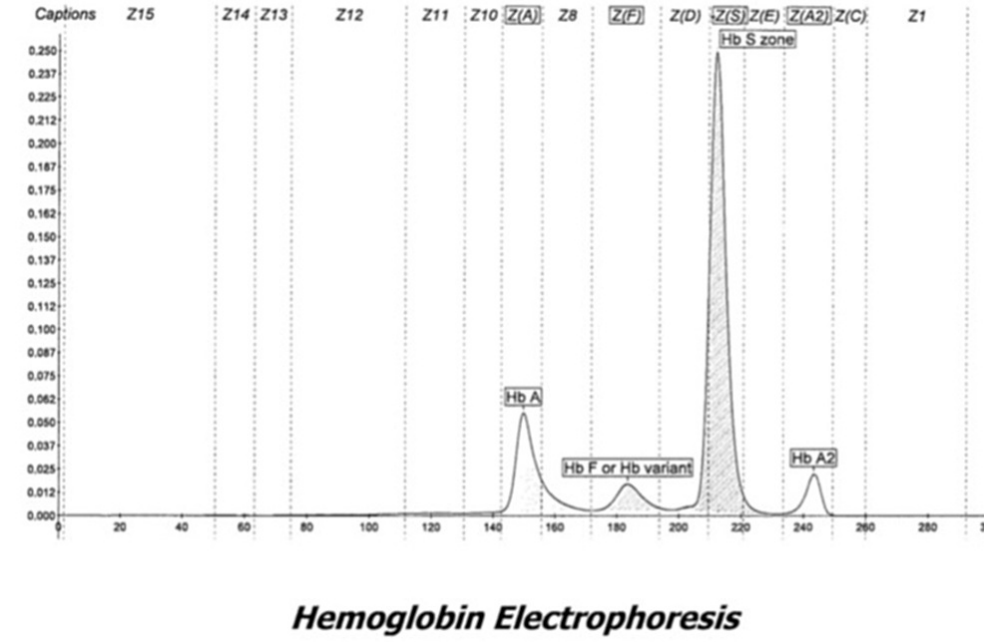
This image represents the specific findings of the hemoglobin electrophoresis completed during the patient presentation. Of note is a highly elevated spike labeled “HbS zone” indicating abnormal levels of sickle cell hemoglobin Hb, Hemoglobin

The patient presented with chronic microcytic anemia which was being treated with iron supplementation as she had iron deficiency as well. However, despite prolonged iron supplementation, her hemoglobin never improved. Physical examination was unremarkable except for “half and half” nails (Figure 1) and upon laboratory testing by high-performance liquid chromatography (HPLC) a low Hemoglobin A (18.2 %), a slightly elevated Hemoglobin F (5.9%), and Hemoglobin A2 (5.7%) with a predominant presence of Hemoglobin S (70.2%) was seen (Figure 2); this was interpreted as the presence of sickle cell/ beta plus thalassemia (Table 1). Complete blood count (CBC) revealed a hemoglobin count of 7.8 g/dl, a mean corpuscular volume (MCV) of 73.5, a reticulocyte count of 2.85%, and hematocrit was 24.9 (Tables 2, 3).

 

 

**Table 1:** Results of the patient’s hemoglobin electrophoresis results at time of presentation, all of which were reported to be in abnormal range. HBG, hemoglobin

Component	Value	Reference range & units
HGB F	5.9 (High)	0.0-2.0%
HGB A	18.2 (Low )	96.4-98.8%
HGB A2	5.7 (High)	1.8-3.2%
HGB S	70.2 (High )	0.0%
HGB Solubility	Positive (Abnormal)	Negative

**Table 2:** Results of the patient’s complete blood cell count taken at time of presentation revealing a microcytic, hypochromic anemia. RBC, red blood cell; HB, hemoglobin; HCT, hematocrit; MCV, mean corpuscular volume

Component	Value	Reference range & Units
RBC	3.39	3.8-5.3 10x6/µL
HB	7.8	11.0-15.0 g/dL
HCT	24.9	35-46%
MCV	73.5	80-100 fL
Platelets	221	130 - 400 10x3/uL

**Table 3:** Results of additional pertinent lab values taken during time of presentation to further investigate the cause for refractory anemia. Of note is the elevated reticulocyte count. LDH, lactate dehydrogenase

Component	Value	Reference range & units
Reticulocyte Percent Auto	2.85 (High)	0.5-2%
LDH	181	125-220 U/L

It is worth notifying that the patient was asymptomatic, specifically, she had never experienced a vaso occlusive crisis. Family history was negative for SCD/sickle cell trait in family members. When the patient was informed about the diagnosis, then she recalled that long ago a doctor had told her about the diagnosis. Management included folic acid supplementation and adequate hydration.

## Discussion

SCD is a common disease seen around the globe inducing multisystem damage in all patients with the disease [1]. Due to its ability to affect multiple systems during disease progression, patients with SCD often face numerous counts of acute illnesses leading to progressive organ damage [1]. SCD is caused by an autosomal recessive mutation on the beta-globin gene on the sixth codon of chromosome 11, inducing an amino acid substitution from glutamine to valine [1]. This mutation leads to the creation of Sickle hemoglobin (HbS), which repeatedly polymerizes red blood cells (RBCs) into a sickle formation during times of low oxygen tension [7]. 

Roughly 10%-15% of SCD is compounded with additionally inherited mutations generating thalassemia phenotypes [8]. Thalassemia is defined as a group of hereditary blood disorders resulting in faulty globin chain synthesis, potentially resulting in insufficient erythropoiesis and increased hemolysis, with eventual bone marrow hyperplasia and skeletal deformities [9]. There are multiple Sickle cell β-thalassemia variants each with differing clinical severity of disease progression and are classified based on the amount of beta-globin produced [3]. Since this patient was able to produce some, albeit reduced amounts of beta-globin, they fall under the sickle cell beta+ thalassemia (HbS/β+ thalassemia) category, a rare genotype [7].

Due to the rarity of this genotype, the clinical presentation of HbS/β+ thalassemia has yet to be fully explored. The literature up to this point suggests that the clinically heterogeneous nature of this variant is quantitatively dependent on HbA levels, with increased HbA levels predicting a milder outcome [4]. The phenotypic classification of these levels are as follows: type I, 1%-7% HbA; type II 7%-14% HbA; and type III 14%-25% HbA [5]. The differing ranges of HbA levels are due to variations in the molecular mutations resulting in the HbS/β+ thalassemia variant. The Type I variant, resulting in the most severe form of the disease, is due to some splice-site mutations of the HBB gene, such as in the β+ first intervening sequence (IVS-I) -110 mutation, occurring within the first intron of the HBB gene [6]. It is thought that the lowered levels of HbA due to this mutation are too low to prevent RBC sickling and propagate more hemolysis and incidences of vaso occlusive crises. This variant is most commonly reported in those from the Mediterranean area [6].

The third type of HbS/β+ thalassemia, with the highest levels of HbA, is due to -88 mutations in the promoter element of the proximal CACCC box [6]. Since this is not a major promoter element associated with the CCAAT or TATA boxes, there is only a mild downregulation of β-globin. This creates a mild phenotype due to enough HbA available to prevent significant sickling and can incidentally cause elevated levels of HbF in some patients [5,6]. Incidentally, this phenotype is most commonly seen in those of African descent [3,6]. Type II HbS/β+ thalassemia is most commonly seen in Greece and is due to a mutation in the IVS-I-110, resulting in an intermediate form of this disease [5].

Due to the slower clinical progression of this subtype, patients can go undiagnosed for several years until complications arise, such as in this patient. Typically, complications of HbS/β+ thalassemia can include refractory microcytic anemia, stroke, avascular necrosis of bones, vaso occlusive crisis, hepatosplenic sequestration, renal failure, or pulmonary hypertension [4,9]. In those with lesser amounts of HbA levels, such as Type I or Type II HbS/β+ thalassemia, these complications may arise in childhood or appear suddenly in adulthood [4]. Interestingly, one study saw that adults are more likely to experience acute chest syndrome in HbS/β+ thalassemia than in other forms of sickle cell β-thalassemia [4]. Acute chest syndrome, while not experienced by this patient, can occur in roughly 50% of patients with SCD, and is defined as an acute non-specific pulmonary illness characterized by new-onset chest pain, cough, tachypnea, dyspnea, increased work of breathing with hypoxia and acute radiographic pulmonary infiltrates [2]. In milder forms of HbS/β+ thalassemia, laboratory values might be more helpful in diagnosis rather than waiting for the emergence of these complications. Typically, these patients will present with hypochromic microcytosis not resolved with iron supplementation, in addition to beta or alpha globin abnormalities, as seen in this patient. Our patient had microcytic hypochromic anemia along with elevated HbS, HbA2 and HbF levels, and lowered HbA levels consistent with a diagnosis of HbS/β+ thalassemia, type III [9]. Additionally, her hemoglobin solubility test, a test commonly performed to identify the presence of abnormal hemoglobin, specifically sickle cell hemoglobin, was positive, indicating a form of SCD was the underlying cause of her microcytic anemia, which would also explain the development of “half and half” nails, a nonspecific finding seen in those with chronic disease [2]. 

Since the clinical presentation of HbS/β+ thalassemia can differ so drastically, treatment can differ on an individualized basis based on clinical manifestations. Hydroxyurea was found to have a beneficial impact on those experiencing recurrent vaso occlusive crises [9]. However, due to the rarity of the disease, there are limited recommendations for the use of hydroxyurea along with the use of red blood cell transfusions or antibiotic prophylaxis. No available management consensus has been reached as to date and is an area needing further evaluation [6]. This patient’s management included cessation of her iron supplementation as iron deficiency anemia was ruled out by her diagnosis. Additionally, she was prescribed a folic acid supplement and advised to regularly hydrate.

## Conclusions

Herein, we have discussed an aged female patient with Sβ+ SCD whose condition was undiagnosed until this encounter. This delay occurred despite the fact she had undergone genetic testing early in her life and that she and her providers had known for years that she co-inherited the hemoglobin S and beta-thalassemia (β+) mutations. Perhaps this could have been prevented by using the term “Sβ+ SCD” for her diagnosis instead of “sickle cell trait beta-thalassemia trait.” This case underscores the need to establish effective educational programs to raise clinicians’ awareness of the diagnosis and management of SCD and its subtypes, evidenced by the fact that all studies conducted on this patient provided clues toward a diagnosis of SCD. Treatment management for all patients with this subtype of SCD is additionally an area for further research.
